# Phthalates Implications in the Cardiovascular System

**DOI:** 10.3390/jcdd7030026

**Published:** 2020-07-22

**Authors:** Melissa Mariana, Elisa Cairrao

**Affiliations:** 1 CICS-UBI, Health Sciences Research Centre, University of Beira Interior, 6200-506 Covilhã, Portugal; melissa.r.mariana@gmail.com; 2 FCS-UBI, Faculty of Health Sciences, University of Beira Interior, 6200-506 Covilhã, Portugal

**Keywords:** plastic contaminants, phthalates, cardiovascular system, blood pressure, atherosclerosis, cardiometabolic risk

## Abstract

Today’s sedentary lifestyle and eating habits have been implicated as some of the causes of the increased incidence of several diseases, including cancer and cardiovascular diseases. However, environmental pollutants have also been identified as another possible cause for this increase in recent decades. The constant human exposure to plastics has been raising attention regarding human health, particularly when it comes to phthalates. These are plasticizers used in the manufacture of industrial and consumer products, such as PVC (Polyvinyl Chloride) plastics and personal care products, with endocrine-disrupting properties, as they can bind molecular targets in the body and interfere with hormonal function. Since these compounds are not covalently bound to the plastic, they are easily released into the environment during their manufacture, use, or disposal, leading to increased human exposure and enhancing health risks. In fact, some studies have related phthalate exposure with cardiovascular health, having already shown a positive association with the development of hypertension and atherosclerosis in adults and some cardiometabolic risk factors in children and adolescents. Therefore, the main purpose of this review is to present and relate the most recent studies concerning the implications of phthalates effects on the cardiovascular system.

## 1. Introduction

According to the World Health Organization (WHO), cardiovascular diseases (CVDs) are the main cause of death globally. Today’s lifestyle and habits, including smoking, sedentarism, and bad eating habits, as well as diabetes and obesity, are considered as the main risk factors for these diseases. However, the influence of environmental contaminants on human health has also been proposed as a cause for CVD, with several studies suggesting a connection between exposure to phthalates and CVDs [1].

Phthalates are normally used in the plastic industry as plasticizers and could be classified as high molecular weight (HMW) and low molecular weight (LMW) [2]. The HMW phthalates are frequently used in food packaging, toys, medical devices, and household products, and include di-(2-ethylhexyl) phthalate (DEHP), the most commonly used phthalate plasticizer for PVC [3,4], butylbenzyl phthalate (BBzP), diisononyl phthalate (DiNP), di-n-octyl phthalate (DnOP), and diisodecyl phthalate (DiDP). Among the LMW phthalates are di-butyl phthalate (DBP), dimethyl phthalate (DMP), diethyl phthalate (DEP), and di-isobutyl phthalate (DiBP), mostly used in paints, adhesives, solvents, personal care products, and medications [2,5]. For a better understanding of the chemical properties of phthalates, these have been extensively presented in Mariana et al. [2]. Phthalates are easily released into the environment during their manufacture, use, or disposal since they are not covalently bound to the plastic [6,7], and, consequently, could be absorbed by the human body through different routes of exposure [8]. They can bind to molecular targets in the body and interfere with hormonal homeostasis [2,9], leading to different disorders in fetuses, infants, children, and adults [10,11], thus being considered as endocrine-disrupting compounds (EDCs). In addition, a common feature of endocrine disruptors is that they do not have a typical dose-response curve, that is, the increase in concentration does not correspond to the increase in their effect, as they present a non-monotonic curve [12]. For this reason, most of the studies performed and described in this review have large concentration ranges and try to cover all situations, daily, chronic, and acute exposure. The maximum concentration described in human urine, for the general population, is 305 µg/L [2,7]. However, it has been reported a concentration in urine between 5–50 mg/L for chronic or clinical exposure [13]. Urinary phthalate measurement has shown that women have higher levels of phthalate metabolites than men, which may be associated with more regular use of personal care products [6]. Also, other studies concluded that children have higher urinary levels of phthalate metabolites (both HMWP and LMWP) than adults, suggesting that they are more exposed to phthalates, and thus, more susceptible to environmental insults [2,5,14,15]. This can lead to adverse health outcomes in later life or adulthood [5,10,16]. In addition, intrauterine exposure to phthalate has also been demonstrated and associated with metabolic disorders that are related to hypertension [5,17]. A positive association has already been shown between phthalates and the development of hypertension [5] and atherosclerosis [18] in adults and some cardiometabolic risk factors in children and adolescents [10,19], but there is still little data on the cardiovascular effects of phthalates. Thus, the main purpose of this review is to present and relate the most recent experimental and observational studies of cardiovascular phthalates effects, both in humans and animals.

The latest studies of the phthalates effects regarding experimental and observational studies on the cardiovascular system will be introduced in this review. For this purpose, a Pubmed search of articles published between the years 2015–2020 was carried out. The database search was performed using a combination of terms related to phthalates (“phthalates”, “phthalates exposure”, “plastic contaminants”) with the cardiovascular system (“cardiovascular system”, “arteries”, “vascular”, “smooth muscle”, “vascular smooth muscle”, “smooth muscle cells”, “endothelium”, “endothelial-dependent”) and cardiovascular outcomes (“cardiovascular diseases”, “myocardial infarction”, “stroke”, “heart failure”, “heart rate variability”, “blood pressure”, “hypertension”, “endothelial dysfunction”, “arteriosclerosis”, “atherosclerosis”, “peripheral vascular disease”). In addition to these terms, we also included in the search EDCs and specific phthalates, and relevant citations were searched in the references of the articles used. From all the articles retrieved, duplicate, unrelated, and inaccessible papers were excluded.

## 2. Animal Studies

### 2.1. Blood Pressure

There are still few studies relating phthalates exposure to CVD in animals, most of which are related to DEHP. Before 2015, it had already been demonstrated that DEHP had adverse effects on cardiomyocyte function of the chick embryo [20] and rat [21,22,23,24], leading to electrophysiological changes on the isolated rat heart [25] and to different effects on blood pressure (BP), increasing systolic and diastolic BP in 33-week-old rat offspring [26], but decreasing the systemic BP due to long-lasting effects on aldosterone levels [27].

Since then, there have been, to our knowledge, six new studies relating phthalates with blood pressure. The first one was performed in 2015 by Lee et al., where 30 mg/kg/day of DEHP were orally administered to female mice, four weeks before copulation, during pregnancy, and lactation until their offspring reached eight weeks old. Then, blood, aortas, white and brown adipose tissue, liver, and brain from the offspring were collected for analysis, concluding that maternal DEHP exposure increased the blood pressure of mice offspring by 20%. The authors suggested that this BP increase may be due to deregulated eNOS (endothelial nitric oxide synthase) activity and increased angiotensin II–AT1R signaling, since there was a reduction in endothelium-derived NO production, which, when compromised, could lead to the development of CVDs, such as hypertension [28,29]. In addition, the offspring aortas showed an increase in the AT1R protein expression (by 2.4-fold), concluding that the binding of angiotensin II to AT1R will lead to vasoconstriction and a consequent increase in BP [29]. Other studies explored oxidative stress markers and cardiac structure in rats’ offspring upon maternal phthalic acid exposure. This study aimed to expose the pregnant female Wistar rats (from day 7 to 16 of pregnancy) to large amounts of phthalic acid. Thus, the compound was added to the diet in different dosages, 1763 mg/kg/day and 2981 mg/kg/day. The authors showed a significant increase in the offspring BP associated with the increased phthalic acid exposure due to cardiac hypertrophy and a significant increase in the thickness of the aorta wall and the descending left coronary artery. It was also demonstrated that phthalic acid significantly increased the oxidative stress and decreased the antioxidant capacity of the cardiac tissue, which, in turn, are related to vascular dysfunction [30]. In 2017, Jaimes et al. showed that mice exposed to DEHP suffered alterations in the heart rate and cardiovascular response. Twelve-week-old male mice exposed to DEHP (1 μg/4 mg/mL) through drinking water for six weeks had a slight non-significant increase in systolic BP and a decrease in heart rate variability. They also observed a significant increase in the endothelin-1, angiotensin-converting enzyme (ACE) and nitric oxide synthase (NOS) gene expression in DEHP-treated animals. These are all associated with the renin-angiotensin system and blood pressure regulation, which may explain these CV alterations [31].

Two different studies performed by the same authors demonstrated that exposure to DEHP and DiNP is associated with increased blood pressure. In the first study, Deng et al. exposed, via gavage, five-six-week-old male mice to different dosages of DEHP (0.1, 1, 10 mg/kg/day) for six weeks [32]. The mice systolic BP measurements showed a significant increase (22%) after 10 mg/kg/day DEHP exposure, which was consistent with the findings of Lee et al. where a 30 mg/kg/day maternal DEHP exposure increased the systolic BP by 20% [29,32]. These results were also proved by the authors through the histopathological analysis, which showed a significant thickening of the interventricular septum and the ventricular wall at the same dosage, which were in accordance with the histopathological results of the Rahmani et al. study [30]. Then, the authors measured the levels of ACE, bradykinin B2 receptor (BK2R), eNOS, bradykinin, Ca^2+^, and NO, all related with blood pressure regulation. Like Jaimes et al. [31], this study also found that DEHP exposure leads to an increase in ACE levels and demonstrated for the first time that it also led to a decrease in bradykinin levels. Knowing that bradykinin is involved in the vasodilation mediated by NO release and could be degraded by ACE, these results demonstrate that the BP increase by DEHP is due to increased ACE levels and inhibition of the bradykinin-NO pathway [32]. Similarly, the same authors analyzed the effects of DINP exposure on blood pressure. The experimental design was identical, differing only in the DINP doses (0.15, 1.5, 15 mg/kg/day) and the use of dexamethasone (DEXA) to build the hypertension model. It was demonstrated that DINP exposure led to an increase in systolic BP, diastolic BP and mean BP, and at 15 mg/kg/day it increased systolic BP by 17.7%, and the heart rate level. Although they are two different phthalates, this is a very similar result to their previous study, with a significant systolic BP increase. After measurement of ACE, AT1R, and eNOS expression in the aorta and NO concentration in serum, the results showed that exposure to DINP or DINP combined with DEXA treatment enhanced the expression of ACE and AT1R and inhibited eNOS expression and NO production, leading to increased BP in mice, which were in accordance with Lee et al. results [29]. However, when the ACE inhibitor (ACEI) was added, it alleviated the increase in BP induced by DINP and DEXA [33].

Lastly, a comparison study between the DEHP and DBP effects in BP was performed by Xie et al. Mice were subjected to intragastric administration of the same doses (0.1, 1, 10 mg/kg/day) of DEHP and DBP for six weeks, and BP was monitored, and only DEHP at 1 and 10 mg/kg/day led to a significant increase in the BP. Deepening the study, the authors found that DEHP exposure increased the levels of ACE and angiotensin II and decreased the NO levels as well as th eNOS expression, but exposure to DBP had no significant changes. On the other hand, there was a significant increase in oestradiol levels after exposure to DBP but not DEHP. The results of DEHP exposure obtained in this study are in agreement with those previously mentioned that also analyzed DEHP effects, leading to increased blood pressure through increased Angiotensin II, ACE, AT1R expression, and reduction of the NO. DEHP and DBP affected the BP differently, partly due to the different oestradiol levels induced by the two phthalates [34].

All of these studies show that DEHP exposure has the ability to elevate BP [29,30,31,32,34], as well as another HMW phthalate, the DiNP [33]. However, the only LMW phthalate studied did not lead to a significant change in blood pressure [34]. Thus, it is possible to hypothesize that the changes in the blood pressure may be due to ACE and AT1R levels increase and inhibition of the bradykinin-NO pathway, and possibly being dependent on the difference of molecular weight of phthalates, but further studies are needed to confirm these hypotheses and to clarify the mechanisms involved.

In order to analyze the molecular pathway induced by the direct effect of DEHP in rat vascular smooth muscle cells, an in vitro experimental study was performed. A range of DEHP concentrations (0.001, 0.01, 0.1, 1, 10, 100 µM) was analyzed in rat aorta through contractility and electrophysiology experiments. The results showed that, at higher concentrations, DEHP induced vasorelaxation in rat aortic rings and inhibition of basal and stimulated calcium current, concluding that this inhibition was responsible for the aorta relaxation [35]. In this sense, this study shows that, in vitro, DEHP has the same effect as the sex hormones in the vascular beds; however, the in vivo effects appear to be opposite to those obtained for oestradiol, so it is necessary to study the chronic exposure of this compound, which can lead to the reversal of the initial vasorelaxant mechanism. Even if the use of the rat aorta as a model is consensual, once it is the simplest way to study the vascular function and the vascular cell signaling [36], this is not deprived of limitations. The study of aortic responsiveness, instead of arteriolar responsivity that is the primary regulator of blood pressure is the main limitation [37], and the use of resistance vessels of the microcirculation should be the next step to study the phthalate cardiovascular effects.

### 2.2. Atherosclerosis

Amara et al. conducted a study to evaluate mice oxidative stress and cardiotoxicity induced by DEHP. Oxidative damage can lead to major lesions in heart tissue, so the authors analyzed the main markers of oxidative stress (malondialdehyde—MDA—and protein carbonyl) in six-week-old male mice treated intraperitoneally for 30 days with different doses of DEHP (5, 50, and 200 mg/kg/day). They found that both markers were significantly increased compared with the control; in fact, heart tissue of myocardial injured mice had elevated MDA levels, suggesting an overproduction of ROS in this tissue. Moreover, the authors also demonstrated that DEHP exposure significantly increased serum lactate dehydrogenase (LDH), aspartate aminotransferase (AST), alanine aminotransferase (ALT), and creatinine phosphokinase (CPK) levels, which are involved in the onset of myocardial damage, and inhibited the cardiac acetylcholinesterase (AChE) activity that may be due to oxidative stress generated by DEHP. This study also demonstrated that DEHP led to changes in the lipid profile, increasing total cholesterol, triglycerides, and LDL-cholesterol levels and decreasing HDL-cholesterol levels, and to an increase in the atherogenic index, increasing the risk of suffering atherosclerosis [38].

An experimental study used smooth muscle cells from rat thoracic aorta (A7r5 cell line) to analyze the influence of different exposure times (up to 48 h) and concentrations of DEHP (2, 3.5, 7, 10.5, 14, 17.5 ppm) on matrix metalloproteinase (MMP)-2 and MMP-9, that are related with atherosclerosis. The results confirmed that DEHP could induce atherosclerosis by significantly increasing MMP-2 and MMP-9 expression through the regulation of different pathways, including p38 mitogen-activated protein kinase (MAPK), extracellular signal regulated kinase 1 and 2 (ERK1/2), Akt, and nuclear factor-kappaB (NF-κB) [39].

Several studies have been using apolipoprotein E-deficient (Apoe−/−) mice as a model for atherosclerosis. This animal model develops all stages of the disease, including the typical human features of dyslipidemia [40]. Therefore, to study the effects of DEHP in the development of atherosclerosis, Zhou et al. resorted to this animal model. In the Zhou et al. study, the six-week-old apoE−/− female mice were exposed to DEHP in drinking water (100 mg/kg/day) for 12 weeks, and the glucose and insulin tolerance, plasma glucose, and triglyceride levels, body weight, and atherosclerotic lesions were analyzed. DEHP had only a small association with blood glucose and glucose tolerance in the early phase of exposure; however, there were no significant differences for atherosclerotic lesions [40]. On the other hand, the study performed by Zhao and collaborators showed opposite results. Serum and heart, aorta, liver, and adipose tissues were collected from five-month-old apoE−/− male mice administered intraperitoneally with 500 mg/kg/day DEHP for four weeks. The results showed that DEHP promoted an enhancement of atherosclerosis due to modifications in cholesterol homeostasis and the deregulation of the inflammatory response [41]. The differences between these two studies may be due to the dose administered; in the second study, it was five times higher, and also in this last study, the animals were older.

These studies demonstrate that DEHP can lead to atherosclerosis in male rats and cell lines since the only study that showed no connection between DEHP and atherosclerosis was performed using female rats. Some possible mechanisms of action of DEHP for the development of atherosclerosis may be due to changes in the lipid profile, which may lead to the appearance of atheromatous plaques, on the other hand, the increase in metalloproteinases also seems to be involved. Nevertheless, more studies should be performed to clarify the effects of DEHP in the development of atherosclerosis, even with more relevant doses to human situations, as well as the mechanisms involved.

Phytochemicals have been proposed as protective compounds of phthalates exposure. Some studies have shown that polyphenols, such as resveratrol, curcumin, apigenin, have the ability to improve or counteract the adverse effects of phthalates, mostly in male reproductive health and cancer [42]. Recent articles have shown that β-thujaplicin, a compound purified from a Taiwan tree (*Chamaecyparis Obtusa*) with several biological activities [43,44,45,46,47,48], could reduce pro-inflammatory cytokine-induced adhesion molecules and block DEHP-induced MMP-2 and MMP-9 expression and activities, which, in turn, lead to the prevention of initiation and development of atherosclerosis [48]. On the other hand, three different studies demonstrated that taxifolin, a flavonoid used in the treatment of cardiovascular diseases, alleviates the effects induced by DEHP in chicken cardiomyocytes. Zhang et al. demonstrated that taxifolin protects chicken cardiomyocytes from DEHP-induced apoptosis by attenuating oxidative stress responses and modulating cytochrome P450 [49]. Cai et al. showed that taxifolin had a protective role by ameliorating DEHP-induced hypertrophy, through the interleukin -6/ Janus kinase/signal transducer and activator of transcription 3 (IL-6/JAK/STAT3) pathway [50], and, lastly, Zheng et al. proved that cardiomyocyte necroptosis and intracellular Ca^2+^ overload, through the CaMKII-RIPK3 signaling pathway, induced by DEHP could be prevented by taxifolin [51].

### 2.3. Cardiac Effects

Four different studies analyzed the cardiac effects of three phthalates and metabolites in mice and zebrafish. In 2018, Tang et al. studied the effect of maternal DEHP exposure on fetal cardiac development in mice. Pregnant female mice were exposed by gavage to different doses of DEHP (0, 250, 500, 1000 mg/kg/day), and the hearts of live fetuses were used for histological analyses and to study several proteins and genes related to transcriptional factors for cardiac development. The results demonstrated fetal cardiac malformation in vivo can occur through maternal DEHP exposure through the inhibition of the expression of transcriptional factors for cardiac development (GATA4/Mef2c/Chf1) [52].

Zebrafish (*Danio rerio*) have been used as a model to study human cardiac diseases since its heart, with one atrium and one ventricle, is one of the first organs to develop and function during embryogenesis [53,54,55]. In 2017, Sun and Liu investigated the developmental toxicity of BBzP in zebrafish embryos’ heart development. From 4 h after fertilization until 72 h (when embryogenesis is complete), the embryos were submitted to different BBzP concentrations (0, 0.1, 0.6, and 1.2 mg/L), and survival, hatching success, growth, malformation, cardiac structure, and function, as well as two genes related to heart development (Nkx2.5 and Tbx5), were analyzed. The results showed that zebrafish embryos exposed to 0.6 and 1.2 mg/L of BBzP had a significant reduction in the heart rate that led to modified cardiac function. BBzP also affected the morphology and normal heart development and downregulated Nkx2.5 and Tbx5 expression in a dose-dependent manner [55]. Similarly, in 2019, Sun and Li studied the toxicity of DBP on zebrafish cardiac development. Like in the previous study, several concentrations of DBP (0, 0.36, 1.8, and 3.6 µM) were used in the same animal model, from 4–72 h after fertilization. Besides reducing blood flow and nutrients supply, that, in turn, led to growth inhibition, DBP also caused pericardial edema, cardiac structure deformities and function alteration, and a significant decrease in heart rate. The same way, Nkx2.5 and Tbx5 expressions were significantly reduced upon DBP exposure in a dose-dependent way [54]. The results of these studies show that both BBzP and DBP induced zebrafish developmental toxicity and affect the embryos heart development, concluding that the heart might be the main target of these phthalates [54,55].

It has been demonstrated that cardiomyocytes of neonatal mice previously exposed to DEHP had significantly altered mRNA levels that could lead to changes in cardiomyocyte structure and arrhythmia, including tubulin, kinesin, TGFβ2 (connexin-43 expression/transport), ryanodine receptor, calsequestrin, calponin, troponin C, cardiac myosin, cardiac calcium transporting ATPase, triadin (calcium handling), voltage-dependent L-type calcium channels, sodium voltage-gated channels, and potassium rectifier channels (ion channels) [23]. In a more recent study by the same research group, the cardiac safety and biocompatibility of mono (2-ethylhexyl) phthalate (MEHP), the main DEHP metabolite, was studied in a clinically relevant concentration (60 µM) through a perfusion system. Langendorff-perfused rat hearts were isolated and analyzed by electrophysiology experiments and optical mapping. The results showed that MEHP-treated hearts had slow atrioventricular conduction, increased atrioventricular and ventricular refractory periods, and also exhibited a triangulated action potential and a steeper electrical restitution curve [56]. These results confirm that DEHP and its metabolite MEHP are associated with the development of arrhythmias.

These studies demonstrate that phthalates have adverse effects on cardiac function in animal models, but there are still few studies on this matter. These studies propose that phthalates are involved in altering the expression of several genes implicated in cardiac development, but the mechanisms remain unclear. Thus, more studies are needed to find out how each phthalate affects the heart in animals, so human investigations on the heart can be developed.

## 3. Human Studies

### 3.1. Blood Pressure

#### 3.1.1. Adults

Of all the risk factors for cardiovascular diseases, blood pressure (BP), as a risk factor or a disease, has been the most studied regarding phthalates implications. Phthalates are suspected to increase BP, in fact, in children and pregnant women, different phthalates have been associated with increased BP, but, in the last years, only one epidemiological study focused on an adult population. This population included 474 female and male participants aged 18–55 years, from China, from whom blood samples were collected and analyzed for 16 different phthalates and lipidic profiles. From all phthalates measured, DBP had the highest concentration, about 59-fold higher than DEHP, but, unexpectedly, only DEHP showed a positive association with systolic BP, without any relationship between diastolic BP and any of the phthalates under study [57].

Despite concluding that exposure to phthalates influences BP in adults, more studies are needed to validate these findings, even with different populations to assess the genetic variability, and mainly to discover the mechanisms associated with the increase in BP. The authors have not proposed a hypothesis for the mechanism by which phthalates lead to hypertension; however, some theories have been proposed. One of the theories, in rats, is that phthalates may lead to hypertension by increasing ACE and AT1R levels and inhibiting the bradykinin-NO pathway. Phthalates may also contribute to insulin resistance, leading to microvascular changes that could result in hypertension. In another perspective, since phthalates have been shown to alter heart rate and vascular contraction, a possible hypothesis may be that they lead to genomic modifications in vasoactive components, such as serotonin or histamine receptors and ion channels, which would lead to increased responsiveness, thus increasing vasomotion and consequent hypertension.

#### 3.1.2. Pregnant Women

For children and pregnant women, there is more evidence of phthalates effects, as they are more vulnerable populations, but even so, there are still few studies on this matter and the conclusions point to different results. One of the studies relating phthalates with BP in pregnancy was performed by Werner and co-workers. Urine samples from 369 American pregnant women were collected at 16- and 26-weeks’ gestation, and the phthalates’ concentration were measured. From the five phthalates analyzed, there was only a significant association between one BBzP metabolite (MBzP) and increased diastolic BP and hypertensive disorders in pregnancy (HDP) [58]. In accordance with the previous study, urinary phthalates concentration has been positively associated with the onset of preeclampsia. According to Peres et al., this multisystemic disease is defined by the development of hypertension in a normotensive pregnant woman after 20 weeks of pregnancy and the appearance of proteinuria or symptoms of target organ injury. It is considered as one of the major causes of maternal and perinatal morbidity and mortality, with significant health risks for the woman and the fetus [59]. A case–control study enrolled 50 pregnant women diagnosed with preeclampsia, from which urine samples were collected four times during pregnancy and nine phthalate metabolites analyzed for each sample. The authors found that DEHP metabolites were significantly associated with preeclampsia throughout the pregnancy, with a potentiated risk closer to the end of pregnancy [60]. However, these results were not consistent with a study carried out by Philips et al. In this epidemiological study, phthalate metabolites’ concentration was measured in a single spot urine sample from 1233 Dutch women in early pregnancy (median gestational age 13.1 weeks). The results showed no association between the 18 phthalate metabolites analyzed with maternal blood pressure or HDP; however, significant associations with placental angiogenic markers were found, supporting that phthalates may contribute to a higher risk for preeclampsia [61].

More recently, there was another study relating different endocrine-disrupting compounds with BP in pregnancy. In this work, 10 phthalate metabolites were measured to 152 pregnant women from three European regions, Spain, France, and Norway. The urine samples were collected three times per day for two weeks (one in the second trimester and another in the third), and the BP was measured at the end of each week. The results showed a decrease in both systolic and diastolic BP upon exposure to some of the phthalate metabolites analyzed, particularly in the second trimester [62]. The authors expected that having less participants in the study but more samples per subject would bring a more efficient outcome compared with studies that gathered more participants but only one single sampling [62], like the study conducted by Philips et al. [61].

In order to analyze the trimester-specific association of phthalates throughout pregnancy, Han and collaborators designed a study to collect one urine sample for each trimester of pregnancy. Each of the 633 participants provided a spot urine sample and blood pressure measurement in each of the three trimesters, and nine phthalates were measured in each sample. Besides, further stratified analyses were performed since it was demonstrated that there might be a variation in maternal BP with different fetus gender [63,64]. The results showed a significant relationship among pregnant women with male fetuses only, with high levels of MiBP exposure in the first trimester associated with increased BP in the second trimester [64].

Despite remaining unknown, several hypotheses have been put forward for the mechanism of increased BP in pregnancy due to exposure to phthalates, including the increase in oxidative stress, which could lead to the release of angiogenic factors [65]; the decrease in serum thyroxin [66], and the increase of inflammatory cytokines [67]. All these may be important factors for gestational hypertension or preeclampsia development [64,68,69,70].

Although the studies presented point to an association between BP in pregnancy and exposure to phthalates, they still have inconsistent results. Only one study showed a decrease in BP, rather than an increase, like most of them presented. Although the purpose of the studies is similar, the investigation design is different, and there are many variables that lead to a difference in the results, from the population size, the number of samples acquired during pregnancy to the genetic variability. So, more studies, including experimental studies, must be carried out in order to have reliable results in this matter, since increased blood pressure during pregnancy may have serious consequences, including hypertension during pregnancy, preterm birth, and later hypertension.

#### 3.1.3. Children

Prevention and control of hypertension have been seen as a health problem that affects only adults; however, recent studies have shown that children with high blood pressure are more likely to become adults with hypertension [71]. Thus, focusing on children and young people, a cross-sectional study performed in 2015 analyzed the association of urinary phthalates with BP, triglycerides and lipoproteins of 1619 US children and adolescents (6–19 years). In this study, DEHP, DiNP, and DiDP were measured, and the results showed that there is a significant association of DiNP and DiDP metabolites with higher systolic BP, with no association detected for triglyceride and HDL [72]. To note, these two phthalates are usually found in cosmetics and personal care products. Another source of phthalates is the medical devices, mainly due to hospitalization situations. Knowing that in those situations, children and infants may be exposed to high levels of DEHP, and that evidence of the link between phthalates and blood pressure in children has been increasing, Jenkins and co-workers decided to explore this issue. Urine samples from about 20 infants were analyzed for DEHP exposure, and idiopathic hypertensive infants were compared with normotensive ones. From the evaluation of the hospital equipment (intravenous fluid bags and respiratory-related tubes) for the presence of DEHP, the authors concluded DEHP exposure is greater in hypertensive infants, and that intravenous exposure is predictive of systolic BP, suggesting that the cortisol/cortisone ratio is implicated in the mechanism [73].

On the other hand, blood pressure and growth of children were assessed after maternal exposure to phthalates during pregnancy, in a Spanish population. Three hundred ninety-one mother-child pairs from a prospective birth cohort study were included, and two spot-urine samples collected in the first and third trimesters of pregnancy were analyzed for phthalate metabolites concentration. The participants were followed for about seven years, and the children’s BP was measured at four and seven years old. The authors concluded that exposure to DEHP metabolites and MEP were associated with lower systolic BP (but not diastolic BP) at four and seven years of age in girls only [74]. In agreement with these results, a very recent study also showed that maternal urine phthalate levels are associated with elevated BP in female offspring [75]. This new study overcomes one of the limitations of the one performed by Valvi et al., increasing the population to 1064 mother-child pairs (from the Netherlands). Phthalate levels were measured in maternal spot urine samples collected three times during pregnancy (one in each trimester), and then the offspring BP was measured at 10 years of age. Analysis was performed for the total group, and for boys and girls separately, and the results showed a positive association between DEHP and DnOP concentrations of the urine samples collected in the third trimester with a decrease in systolic and diastolic BP in girls. Again, these results prove that there is a sex-specific difference in the association of exposure to phthalates during pregnancy and BP [75]. In a large observational study, relating a series of pre- and post-natal environmental exposures (from chemicals like phthalates to air pollution, traffic, and lifestyle) to blood pressure in children, mono-benzyl phthalate (BBzP metabolite) was associated with a decrease in BP. The results obtained are from 1277 children (age 6–11 years) from the European HELIX (Human Early-Life Exposome) project that gathered data from six cohorts from different countries (United Kingdom, France, Spain, Lithuania, Norway, and Greece). A wide range of exposures was evaluated in the HELIX project, pregnancy blood and urine samples were stored, and the systolic and diastolic BP were measured from the children [76]. Besides its large number of participants, the sample size was very small, taking into account the various exposures investigated. Either way, this study is in agreement with the ones performed by Valvi et al. and Sol et al., that also reported a decrease in systolic BP [74,75], but there was no evidence regarding the sex-specific differences. It is worth noting that the sex-specific effects of phthalates have already been presented in the study from Han et al., where they also showed that MiBP exposure is associated with increased BP in women pregnant but with male fetuses only [64]. Thus, this topic must be intensively studied to understand whether the effect of phthalates exposure on blood pressure is dependent on the child’s sex as well as the underlying mechanisms and long-term consequences. It would also be of interest to resort to measurements of phthalate exposure in the children themselves to compare with those of the mothers.

From the previously mentioned investigations, there are inconsistent results, with some relating phthalates to increased BP and others to a decrease in the BP. Among these differences, similar parameters were found between the studies. When the phthalate concentration is measured in the children themselves, it appears that they show an increase in systolic BP; however, when the relationship between the mother and child is analyzed, by measuring maternal exposure and comparing with BP in children (aged 4–11 years), these have a decrease in the BP. This difference has not yet been explained, and the discrepancy in the results may be due to genetic diversity; however, further studies are needed to understand how phthalates are affecting the BP in children.

### 3.2. Atherosclerosis

The relationship between human phthalate exposure and atherosclerosis has been poorly studied. In fact, in addition to the study carried out in 2011 by Lind and Lind, demonstrating that there is a relationship between phthalates and atherosclerotic plaques in an elderly population [18], only three more observational studies were published. Through the same prospective study used by Lind and Lind (2011)—the Prospective Investigation of the Vasculature in Uppsala Seniors (PIVUS) study—Wiberg et al. analyzed the link between MBzP, the main metabolite of BBzP, circulating levels, and atherosclerosis in the carotid arteries. From a population of 1003 people (aged 70), the levels of MBzP were measured in serum, and related to the prevalence of carotid atherosclerotic plaques, intima-media thickness, and echogenicity of the intima-media complex. The authors found MBzP has a role in the development of atherosclerosis due to a strong association with all the subclinical parameters analyzed [77]. From a different perspective, a case-control study aimed at analyzing the association between phthalate exposure and risk of coronary heart disease also found a link to atherosclerosis. Urinary phthalate metabolites from middle-aged workers, mostly males, with a history of acute myocardial infarction or angiography-documented severe coronary heart diseases, were measured, as well as novel atherothrombotic markers, including high-sensitivity C-reactive protein (hs-CRP), fibrinogen, and D-dimer. The authors found a positive association between DEHP and DBP metabolites and coronary heart disease, and the increased levels of the atherothrombotic markers suggest that the inflammatory and hemostatic pathway could be the link between phthalate exposure and the risk of subclinical atherosclerosis, enhancing the risk of coronary heart disease [1]. In the same year, Su and co-workers analyzed how the cardiovascular health of a young population is affected by the constant exposure to plastic and, consequently, to phthalates. Seven hundred eighty-seven participants (with a mean age of 21 years old) were recruited for the study and separated into two groups, with elevated BP and normal BP. Urine and blood samples were collected, and subclinical atherosclerosis assessed through the measurement of carotid intima-media thickness. Urinary metabolites of DEHP and DBP were found to be significantly associated with carotid intima-media thickness, an indicator of the development of atherosclerosis. Besides, body mass index, diastolic BP, hypertension, diabetes mellitus, and levels of fasting glucose and triglycerides were all associated with increased levels of MEHP, suggesting that exposure to DEHP is linked to several cardiovascular risk factors that can lead to atherosclerosis [78].

Using human THP-1 macrophages, Wang and collaborators investigated the link between exposure to DBP and the development of atherosclerosis, analyzing the lipid metabolism. They found that there was a non-linear inverted U-shaped relationship between DBP and lipid accumulation since when the THP-1 macrophages were exposed to 10^−7^ mol/L of DBP, there was an increase in the lipid accumulation, by inhibiting cholesterol efflux through ABCA1 expression suppression. This may enhance the development of atherosclerosis [79]. From another perspective, the DEHP effect was analyzed on the release of tissue factor-bearing microparticles (TF-MPs). The generation of endothelial MPs is promoted by plasminogen activator inhibitor type 1 (PAI-1), and the procoagulant activity of TF-MPs is crucial for vascular hemostasis. M1 and M2 macrophages, which have been linked to the progression of atherosclerosis, were incubated with different concentrations of DEHP (0, 10, 20, 50, 100, or 200 nM) for 24 h, and several protein assays were performed. The authors discovered that after M1 macrophages DEHP exposure, there was a significant increase in transforming growth factor-β1 (TGF-β1) protein production, TF protein levels in culture supernatants, and PAI-1 expression, that, upon Smad2, Smad3, or Smad4 silencing, was attenuated, as well as the TF-release from macrophages. Concluding that, DEHP leads to TF-MPs formation in human M1 macrophages through TGF-β1/Smad/PAI-1 signaling pathway [80].

Although the study populations in these three new observational reports are all from different age groups, they all corroborate that exposure to phthalates, mainly DEHP and DBP, has adverse cardiovascular health effects, enhancing the development of atherosclerosis. However, the data is still limited, and new longitudinal and experimental studies are needed to validate and raise awareness for phthalates effects, since only two experimental studies have been reported, and the mechanism associated with the development of atherosclerosis is still unclear.

### 3.3. Cardiometabolic Risk and Metabolic Syndrome

Metabolic syndrome (MetS) is a cluster of several cardiometabolic risk factors, including insulin resistance, dyslipidemia, hypertension, and obesity [81], and some studies have already shown the association between these cardiometabolic risk factors and phthalate metabolites [22,82,83,84,85]. In addition to the studies already described that reveal the link between phthalates with BP and atherosclerosis, other recent studies show the association between this endocrine disruptor and cardiometabolic risks, from an early age to elderly.

In a cross-sectional study performed by Kataria et al., 41 healthy children (10–13 years old) from New York, USA, provided the first-morning urine sample to analyze the association between urinary phthalate metabolites and oxidative stress, insulin resistance, body mass, and endothelial dysfunction. Upon urinary phthalate metabolites measurement as well as oxidative stress, insulin resistance, and biomarkers of blood pressure and vascular function. The authors found an association between HMW phthalate metabolites and increased body weight and risk of insulin resistance, and also a positive correlation between DEHP metabolites and F2-isoprostane levels (systemic oxidative stress biomarker), which leads to the conclusion that phthalates could cause changes in metabolic and oxidative stress profiles and disturbances in the vascular function of healthy children [86]. In children and adolescents, the great increase in obesity observed in recent years has caused a lot of concern about their cardiovascular health. Gathering that exposure to phthalates has also been associated with cardiometabolic risk factors, some authors have tested that hypothesis. In 2018, Amin and collaborators investigated the association of phthalate metabolites concentration in urine with obesity indices and BP in a pediatric population. Urine and blood samples were collected from 242 children and adolescents (6–18 years old) from Iran, for measuring urinary phthalate metabolites and the lipidic profile, respectively. A physical examination (weight, height, waist circumference (WC), and BP measures), and a questionnaire were also applied to the participants. The authors found that these children have high exposure to phthalates since MBzP, MBP, and MMP were found in all the participants and MEHP, MEOHP, and MEHHP were observed in 99.6%, 87.85%, and 29.26% of the participants. The levels of phthalate metabolites were different in all weight groups, being significantly higher in obese children than overweight or normal children, concluding that there is an association between phthalate metabolites with obesity and increased BP in this population [87]. The following year, Mansouri et al. published a case-control study, also conducted in Iran, including 320 participants (7–18 years old) of a national school-based surveillance program. Like in the previous study, urine and blood samples were collected and a physical examination and a questionnaire were also applied to the participants, and the concentration of phthalate metabolites measured was compared between those with and without overweight, and those with and without cardiometabolic risk factors (80 in each group). With this study, the authors found an association between MBP and MEHP concentrations and increased cardiometabolic risk factors in normal-weight children, as well as higher concentrations of MEHP increased the risk in those with overweight, concluding that the phthalate-induced cardiometabolic risk is independent of the weight status in children and adolescents [88].

The cardiometabolic risk factors have also been analyzed in a Serbian male population. These 102 volunteers, aged 18–55 years, were divided in normal weight, and overweight and the first-morning urine and blood samples were obtained after 12h of fasting. Then, the relationship of phthalate metabolites concentration with anthropometric and biochemical parameters was analyzed. The results demonstrated that overweight participants had significantly high MEP levels in urine compared to normal ones, which could be related to increased triglycerides and decreased HDL cholesterol levels in serum. Also, phthalate urine levels were associated with insulin resistance, while increased alanine aminotransferase (ALT) and aspartate aminotransferase (AST) serum levels and body mass index were found to be related to phthalates exposure [89].

Oxidative stress has been pointed out as a possible mechanism for cardiometabolic risk induced by phthalates, for that very reason, Dong et al. decided to explore this issue in a diabetic population from Shanghai. Three hundred volunteers over the age of 50 entered the study, where phthalate metabolites were measured in urine, biomarkers of oxidative stress in serum and insulin resistance risk was assessed by substitute indices. A positive association was found between exposure to phthalates and γ-glutamiltransferase, and with oxidative stress biomarkers (8-hydroxy-2′-deoxyguanosine and malondialdehyde), that can lead to insulin resistance development [90].

James-Todd et al. gathered data from 2719 participants (aged 20–80 years) from the National Health and Nutrition Examination Survey (NHANES) 2001–2010. This study revealed a correlation between the DEHP metabolites concentration and the incidence of obesity, the decrease of HDL-cholesterol levels and the incidence of hypertension. Higher urinary concentrations of DEHP metabolites were associated with increased odds of developing MetS in men, while in women (pre-menopause), MetS was associated with higher concentrations of MBzP [91]. In a cross-sectional study, data from 5251 adult South Koreans were analyzed for urine phthalate metabolites levels and metabolic syndrome parameters. The authors analyzed the link between MetS and several DEHP metabolites, but only found an association with the sum of DEHP metabolites and MEHHP [92].

Despite all the limitations that these studies presented, from small population size to analysis in a one-time sample measure, all of them correlated exposure to phthalates with cardiometabolic risk and (consequent) metabolic syndrome in all age groups.

It has been proposed that oxidative stress is involved in the mechanism by which phthalates lead to cardiometabolic risk, and that these compounds could obstruct lipid and glucose homeostasis, induce insulin resistance leading to increased diabetes and CVD risk, as well as that phthalates affect the production of androgen hormones that also lead to diabetes, CVD, metabolic syndrome [82,83,84,89,90,93]. However, there are still few studies on this matter, mechanisms involved must be discovered. Further longitudinal studies are needed to evaluate the clinical effects of these findings, with a larger population size and several biological samples collected at different time-points.

### 3.4. In Vitro Studies

Besides all these observational studies, there has been an increase in experimental research in humans. There has been evidence that low concentrations of MEHP can induce apoptosis through a mitochondrion-dependent pathway mediated by reactive oxygen species (ROS) in human umbilical vein endothelial cells (HUVEC) [94]. Two other studies continued to focus on the effects of MEHP in human endothelial cells, Wu and co-workers concentrated on the cytotoxic effects of MEHP and the relationship between autophagy and apoptosis [95], whether Liu et al. investigated the oxidative stress pathway in the autophagy induced by MEHP [96]. As it was previously stated, MEHP could induce apoptosis through a mitochondrion-dependent pathway, but the exact mechanisms and the coordination of apoptosis and autophagy in cardiovascular cell fate needs to be fully understood. Using the human endothelial cell line EA.hy926 treated with MEHP (200 µM), Wu et al. detected an induction of autophagy in the early stage of treatment and at 12 h-treatment, there was an increase in the levels of apoptosis, mitochondrial outer membrane permeabilization, and lysosomal membrane permeabilization. The authors proposed that autophagy activated by MEHP is an upstream event triggering lysosomal membrane permeabilization that would lead to apoptosis through the lysosomal-mitochondria pathway [95]. The same way, EA.hy926 cells were treated with several concentrations of MEHP (0–200 µM) for 24 h and the results demonstrated that MEHP induced an increase in the autophagosome number in a dose-dependent way and caused autophagic cell death, with ROS playing a significant part, since the addition of a ROS inhibitor protected against MEHP cytotoxicity and decreased LC3-II (a soluble protein recruited to autophagosomal membranes) protein expression. These findings suggest that after exposure to MEHP, the cells suffer autophagy elicited by ROS through the Akt1 pathway, a major regulator of autophagy [96].

Using the HUVEC cell line, Yang et al. conducted a study to investigate the protective role of 6-gingerol against MEHP effects. They investigated if 6-gingerol, ginger’s most pharmacologically active compound considered as an antioxidant, could inhibit MEHP induced oxidative stress that could reduce DNA damage. For this, after a 1 h incubation with 6-gingerol, different concentrations of MEHP (0, 10, 20, and 40 µM) were added for 24 h, and it was showed, through the comet assay, that 6-gingerol could prevent MEHP induced DNA strand breaks. They also showed that MEHP led to increased intracellular ROS that was reduced after the addition of 6-gingerol. To corroborate these results, 6-gingerol prevented the glutathione (an intracellular antioxidant molecule) depletion, that led to ROS quenching and protection of cells from toxic compounds. In addition, 6-gingerol also blocked the ATM/Chk2/p53 pathway, which is involved in DNA damage. Overall, 6-gingerol has great potential in the therapy of MEHP toxicity [97]. All these studies point out, that MEHP leads to apoptosis, and provides information about the underlying mechanisms. A possible mechanism may be through the increase of autophagosomes that could be mediated by ROS in a mitochondrial-dependent manner, and MEHP effects could be inhibited by decreasing ROS with 6-gingerol. Either way, more studies are required to fully understand the mechanism involved.

In a different study, Posnack et al. evaluated DEHP effects on calcium handling of human embryonic stem cell-derived cardiomyocytes. The conditions used in this study, regarding concentration (5–50 μg/mL) and duration (24–72 h) of DEHP exposure, are the same as clinical exposure conditions, revealing that DEHP had negative chronotropic and inotropic effects and reduced intercellular connectivity of cardiomyocytes. Also, DEHP reduced the calcium transient amplitude and duration, and reduced the spontaneous beating rate, affecting cardiomyocyte contraction [13].

Overall, more experimental studies are needed to fill in some gaps on the information already obtained, in this case regarding the MEHP mechanism, but mainly to cover other phthalates and their metabolite effects, as well as using different cell types related to the cardiovascular system and different times and concentrations of exposure.

## 4. Conclusions

This work provided a comprehensive summary of phthalates effects on the cardiovascular system, describing the latest publications from human and animal studies. From the concepts used in the database search, the most studied so far are blood pressure, atherosclerosis, and cardiometabolic risk factors, that in turn, could lead to the development of CVD, including hypertension, heart disease, and stroke.

When it comes to blood pressure, studies in animal models showed that HMW phthalates could lead to increased BP, while LMW phthalates have no effect. In human studies, the most studied populations are those considered more vulnerable, which are the children and pregnant women. In these, the majority of the studies found an increase in BP for different phthalates; however, when comparing phthalate levels in the mother’s urine with the children’s BP, the results revealed a decrease in BP. This is an interesting result, but it still has to be proved by other studies and to be explained. Although most of the studies presented have shown that several phthalates are associated with elevated blood pressure, the mechanisms involved are unclear. Several hypotheses have emerged for the possible mechanism involved, including oxidative stress, immune and inflammatory responses, impaired angiotensin-aldosterone system.

Few studies have also analyzed phthalates cardiac effects, but only on animal models. The results of those studies demonstrated that in rat DEHP induced fetal cardiac malformation in vivo, and both DEHP and MEHP are associated with the development of arrhythmias; in zebrafish embryos, the heart might be the main target of BBzP and DBP, affecting its development.

Both in animal and human studies, it was found that phthalates can lead to atherosclerosis. In animal studies, possible mechanisms have been suggested, from oxidative stress to modifications in cholesterol homeostasis and deregulation of the inflammatory response. It has been proved that some compounds β-thujaplicin could prevent the initiation and development of atherosclerosis and taxifolin can protect chicken cardiomyocytes apoptosis, hypertrophy and necroptosis induced by DEHP. Three new human studies agreed that exposure to phthalates enhances the development of atherosclerosis in different age groups; however, the data is still limited, and new longitudinal and experimental studies are needed to validate these results as well as the mechanisms involved in animal studies that can be extrapolated to humans.

Besides BP, other cardiometabolic risk factors have been under study and correlated to exposure to phthalates, which can lead to metabolic syndrome in all age groups. It has been proposed that phthalates can obstruct lipid and glucose homeostasis, induce insulin resistance leading to increased diabetes and CVD risk, affect the production of androgen hormones that will also lead to diabetes, CVD, metabolic syndrome, and that oxidative stress is involved in these processes. Further studies are needed to elucidate the mechanisms involved in the development of cardiometabolic risk by phthalates. Regarding human in vitro studies, the results showed that MEHP leads to apoptosis, possibly due to increased autophagosomes mediated by ROS in a mitochondrial-dependent manner, in human endothelial cells. In human embryonic stem cell-derived cardiomyocytes, DEHP had negative chronotropic and inotropic effects and reduced the calcium transient amplitude and duration.

From all the observational studies, it is worth noting that the discrepancies between the studies may be due to different study designs. The investigation’s purpose has to be different, and there must be homogeneity of the population and the samples since some of them collect only one single sample and others several samples for a period of time. This latter would be the best option, so exposure to phthalates would be analyzed over time. Also, there are still very few experimental studies in this area, and the in vitro experiments are very scarce using different types of cells. The results obtained would have to be corroborated by other investigations, so the mechanisms involved are discovered, and the exposure to other phthalates should also be analyzed. Besides all these studies demonstrating that phthalates have adverse effects on cardiovascular health, it is important to mention that they are not associated with CV-related death [98].

This review of the recent literature suggests that early life and adult exposure to phthalates may contribute to adverse cardiovascular health, with changes in BP and risk of atherosclerosis. With this evidence, it is important to inform the population about these risks and to promote public health. In order to do that, sources and routes of phthalate exposure must be of public awareness so they can be greatly reduced, especially in vulnerable populations, pregnant women, children, and the elderly.
